# Cone-beam computed tomography study of the incidence and characteristics of the second mesiobuccal canal in maxillary permanent molars

**DOI:** 10.3389/fphys.2022.993006

**Published:** 2022-11-24

**Authors:** Yuan-Qing Xu, Jie-Qi Lin, Wei-Qun Guan

**Affiliations:** ^1^ Department of Stomatology, Union Hospital, Fujian Medical University, Fuzhou, China; ^2^ School of Stomatology, Fujian Medical University, Fuzhou, China

**Keywords:** cone-beam computed tomography, three-dimensional imaging, maxillary molar, second mesiobuccal canal, root canal therapy

## Abstract

**Objective:** This study aimed to review the characteristics of the second mesiobuccal canal (MB2) in the maxillary first and second permanent molars of 500 patients to analyze the incidence of MB2 and its connection with gender, age, tooth position, and mesiobuccal (MB) root length. The study also aimed to investigate the distribution of the root canal orifice on the pulp chamber floor to obtain an imaging reference for clinical practices related to maxillary molars in Fujian, China.

**Methods:** In accordance with the enrollment criteria, cone-beam computed tomography images of the maxillofacial area were collected from 500 patients, including 1,000 maxillary first molars and 1,000 maxillary second molars. The patients were grouped according to gender and tooth position. The incidence of MB2 was observed, and the MB root length and distribution angle of the mesiobuccal-palatal-distobuccal root canal orifices were measured for statistical analysis.

**Results:** The overall incidence of MB2 in maxillary first and second molars was 51.1% and 32.9%, respectively. The incidence of MB2 in maxillary molars was 50% in male patients and 35.45% in female patients, with a significant difference (*p* < 0.05). The incidence of MB2 in maxillary molars was significantly higher in subjects aged below 50 (especially in male patients) than those aged 50 and above. The MB root length of maxillary first and second molars with MB2 was longer than that without MB2, and the difference was statistically significant. An association was identified between the incidences of MB2 in contralateral molars of the same type and in ipsilateral adjacent molars. When MB2 occurred in the MB root of maxillary molars, the root canals were mostly classified as type IV, followed by type II. The angle formed by the MB-P-DB orifices on the pulp chamber floor in the maxillary first and second molars was (25.23 ± 5.20) and (20.17 ± 10.88)°respectively.

**Conclusion:** The incidence of MB2 in maxillary molars is high in Fujian, China. The occurrence of MB2 is affected by gender, age, and length of MB root, and it occurs symmetrically in adjacent molars and in contralateral molars of the same type. In addition, the distribution law of the main root canal orifice at the pulp chamber floor is conducive to locating MB2, thereby guiding clinical operations.

## 1 Introduction

To achieve clinically successful therapy, all root canals require thorough preparation, debridement, and filling (Vertucci, 1984). Studies have shown that missed treatment is very common for second mesiobuccal canals (MB2s) in maxillary molars because they are difficult to detect, resulting in the highest failure rate of root canal therapy (Karabucak et al., 2016). For this reason, the exact positioning and treatment of MB2 hold the key to a long-term therapeutic effect.

According to the *Guidelines for radiographic examination in cariology and endodontics*, X-rays and panoramic X-rays are the first choice for conventional endodontic treatment (Society of Cariology and Endodontics and Chinese Stomatological Association, 2021). But traditional periapical and panoramic films can only present 2D imaging of 3D structures. Some scholars also believe Micro-Ct of high resolution, which can show the adequate visualization of tooth internal structures (Putignano et al., 2021). However, due to its high dose of radiation is not suitable for clinical practice, whereas cone-beam computed tomography (CBCT) can clearly display the 3D structures of internal tissues of the tooth, thus offering a better understanding of the anatomy of the root canal (Venskutonis et al., 2014). For this reason, this technology has gradually been popularized and applied in the field of stomatology.

The global incidence of MB2 in maxillary first molars varies greatly from 36.3% to 97.6% (Pan et al., 2019; Al Mheiri et al., 2020; Martins et al., 2020), and that in maxillary second molars is between 8.5% and 53.14% (Lin et al., 2017; Candeiro et al., 2019; Aydin, 2021; Faraj, 2021). Such a difference suggests that ethnicity, dietary habits, and geographical environment in different countries and regions may affect the morphological development of root canals. Compared with previous relevant studies in Chinese population mostly limited to single maxillary molar (Wu et al., 2017; Zhang et al., 2017), the present study observed the characteristics and relationship of MB2 occurrence between the maxillary first and second permanent molars comprehensively. In addition, because of the common characteristics of dentin deposition during root development and MB2 formation, we first proposed a correlation between MB root length and the incidence of MB2. The null hypothesis is that the MB root length distribution with and without the MB2 group is not statistically significant. The present study carried out a retrospective analysis of the CBCT images from 500 patients in the region to review the characteristics of MB2 in the maxillary first and second permanent molars to analyze the incidence of MB2 and its connection with gender, age, tooth position, and MB root length. The study also aimed to provide a reference for clinical treatment in Fujian, China.

## 2 Materials and methods

### 2.1 Data acquisition

This study retrospectively analyzed the imaging data of patients who underwent CBCT due to impacted wisdom tooth extraction, orthodontic treatment, or implant surgery.

Inclusion criteria: 1) complete maxillary first and second molars; 2) the root apex was fully developed and free of periapical periodontitis and other diseases; 3) there were no root canal filling materials, posts, or crown restorations; 4) CBCT images were clear, without distortion or overlap.

The required sample size was calculated based on the primary outcome of this study (MB2%) using PASS 15 software (NCSS, Kaysville, Utah, United States). According to previous studies, the incidence of MB2 of the maxillary first and second molar is 69.6% and 39% (Martins et al., 2020). With 90% power using 2-sided tests at the 0.05 level, entering Tests for Two Proportions can be counted as 54 cases per group were needed, considering an anticipated dropout rate of 20%, the finale sample size were defined as at least 136 cases were enrolled. A total of 500 patients were collected finally.

In accordance with the inclusion criteria, a total of 500 patients (225 male patients and 275 female patients) who visited the Stomatology Department of Fujian Medical University Union Hospital and underwent CBCT in the hospital’s Department of Oral Radiology between April 2020 and September 2020 were enrolled. These patients were aged between 13 and 74, with an average age of 32.71 ± 13.46. The study was approved by the Ethics Committee of Fujian Medical University Union Hospital (approval number: 2022KY086).

### 2.2 Instruments

The scanning instrument used in this study was the Carestream CS9300 Select (Carestream, US), with imaging parameters set as: medium field of view (field width and height were both 10 cm), scanning tube voltage 90 kV, tube current 4 mA, scanning duration 8.01 s, and slice thickness 180 µm. The CBCT images were captured with the patient in an upright standing position, and all imaging was conducted by the same full-time radiologist.

### 2.3 Image analysis

Using CS 3D Imaging software, the same trained radiologist responsible for film reading first randomly selected 20 CBCT cases of maxillary first and second molars, observed the data, and carried out a paired sample rank-sum test, before determining the reliability of observer agreement. According to the calculations, *p* = 0.62 > 0.05, indicating that there was no statistical difference between the two measurements. The radiologist then performed analysis in the same space and with the same instrument. In this process, the observer is not aware of the basic information such as the age and sex of the patient, thereby reducing the bias.

**FIGURE 1:**
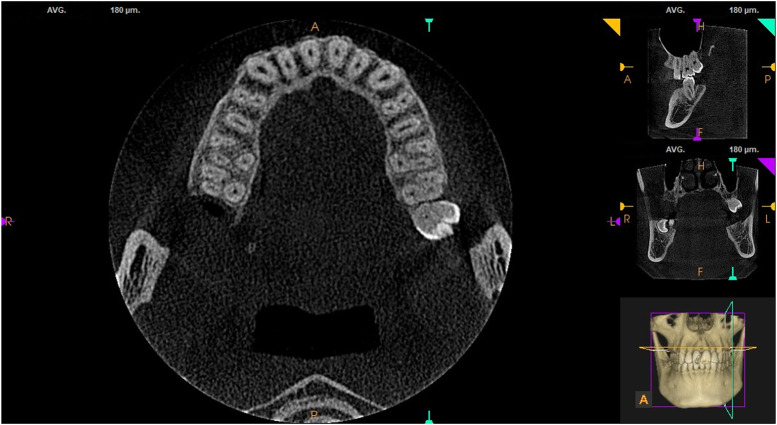
MB2 in maxillary first and second molars.

All CBCT images were taken from the pulp chamber floor to the root apex. The images were observed by moving the roller continuously in the sagittal, coronal, and axial planes. Each tooth measured three times, and the average value was taken. The contents analyzed consisted of the presence or absence of MB2 in maxillary first and second molars (see Figure 1), the mesiobuccal-palatal-distobuccal (MB-P-DB) angle of the pulp chamber floor, and MB length. The software with its own tools for measuring length and angle. Measuring MB root length on the sagittal plane with reference to the distance from cemento-enamel junction (CEJ) to the physiological apical foramen, and locating the center of each root canal orifice on the horizontal axial surface of the pulp chamber floor to measure the three-point angle. Root canal morphology is divided into 8 classes according to the Vertucci’s classification (Vertucci, 1984). Type I (1-1)– From A single tube with a medullar cavity extending to the tip of the root; Type II(2-1) – At the base of the medullary cavity, the two tubes are separated and the near root tip is fused into one root tube; Type III(1-2-1) – a single tube leaves the bottom of the medullary cavity, with two tubes in the middle, and finally a single tube leaves the root foramen; Type IV(2-2) – two Independently separated single tubes, extending from the medullary cavity to the tip of the root; other types are relatively rare. A month later, the radiologist measured all the images again, and the mean of the measurements obtained in the two phases was used for final analysis.

### 2.4 Statistical analysis

Data were statistically analyzed using SPSS (version 25.0; SPSS IBM, Armonk, New York, United States), with measurement data represented as (‾x ± S) and enumeration data as rate. The incidence of MB2 in different age groups and tooth positions was compared using a χ^2^ test. The samples were grouped based on the presence or absence of MB2, and Mana-Whitney test (the sample does not conform to normal distribution) was carried out to statistically analyze whether there was a difference in MB root length. The relationship between the parameters is tested by spearman rank correlation. All tests are bilateral, with a test level of α = 0.05 and *p* < 0.05 were considered statistically significant.

## 3 Results

### 3.1 Overall incidence of MB2 in maxillary first and second molars

This study included CBCT images from 500 patients, 225 of whom were male and 275 of whom were female, aged (32.71 ± 13.46) years. The male patients were aged (33.20 ± 13.98) years and the female patients (32.31 ± 13.03) years. The study included a total of 1,000 maxillary first molars and 1,000 maxillary second molars, of which 511 maxillary first molars and 329 maxillary second molars had MB2, indicating that the overall incidence of MB2 in maxillary first molars and maxillary second molars was 51.1% and 32.9%, respectively.

### 3.2 Factors influencing MB2 occurrence

#### 3.2.1 Gender

Further analysis based on gender showed that the average incidence of MB2 was 50% in male patients and 35.45% in female patients (42% overall). The χ^2^ test showed that, in terms of the tooth position of maxillary first and second molars, the incidence of MB2 in males was significantly higher than that in females (see Table 1; *p* < 0.05).

**TABLE 1:** MB2 in maxillary first and second molars between the female and the male in FuJian area.

Tooth position	Number (n)		
	(Proportion %)		
	Male	Female	Total
Tooth 16	128 (56.89%)*	125 (45.45%)*	253 (50.6%)^@^
Tooth 26	128 (56.89%)*	130 (47.27%)*	258 (51.6%)^@^
Tooth 27	91 (40.44%)*	71 (26.10%)*	162 (32.4%)^@^
Tooth 17	103 (45.78%)*	64 (23.27%)*	167 (33.4%)^@^

* The incidence of MB2 in the maxillary first and second molars of the same name was higher in males than in females (*p* < 0.05).

@The incidence of MB2 in the left and right maxillary molars of the same name was of no statistical difference (*p* > 0.05).

#### 3.2.2 Tooth position

The incidence of MB2 was 50.6% in tooth 16, 51.6% in tooth 26, 32.4% in tooth 17, and 33.4% in tooth 27. The incidence of MB2 in the left and right maxillary molars of the same type was similar (see Table 1), without statistical difference (*p* > 0.05). Spearman’s correlation analysis revealed that there was a positive correlation between MB2 incidence in paired maxillary first and second molars. According to the coefficient, when MB2 occurred in tooth 16, tooth 26 was the most likely to also have MB2, followed by tooth 17 and tooth 27. This shows that MB2 is most likely to occur in the contralateral tooth of the same type, followed by the ipsilateral adjacent molar and the contralateral tooth of a different type. This rule also applies to the remaining maxillary first and second molars (see Table 2).

**TABLE 2:** Spearman’s correlations of MB2 incidence of different maxillary molars in Fujian area.

Group	Tooth 16			Tooth 26		
	Tooth 26	Tooth 17	Tooth 27	Tooth 16	Tooth 17	Tooth 27
Correlation coefficient	0.75*	0.54*	0.40*	0.75	0.47	0.49
*p*	<0.0001			<0.0001		
Group	Tooth 17			Tooth 27		
	Tooth 16	Tooth 26	Tooth 27	Tooth 16	Tooth 26	Tooth 17
Correlation coefficient	0.54	0.47	0.66	0.40	0.49	0.66
*p*	<0.0001			<0.0001		

*Spearman correlation analysis revealed that if MB2 exists in a tooth position in maxillary first and second molars, it is the most likely to occur concurrently in the contralateral tooth of the same name, followed by the ipsilateral adjacent molar, and then the contralateral tooth of a different name.

#### 3.2.3 Age

Subjects enrolled in the study were aged 13–74 years, with 64 aged younger than 20, 195 aged 20–29, 113 aged 30–39, 54 aged 40–49, and 74 aged 50 and above. The incidence of MB2 in maxillary first molars in each age group was 57.03%, 52.31%, 55.75%, 54.63%, and 33.11%, respectively, and that in maxillary second molars was 49.22%, 32.05%, 46.28%, 30.56%, and 17.57%, respectively. The incidence of MB2 in maxillary first molars was always higher than that in maxillary second molars, regardless of age group. Multiple χ^2^ comparison following correction of MB2 incidence in maxillary first and second molars in different age groups concluded that the incidence of MB2 in maxillary first molars was significantly higher in patients aged below 50 than in those aged 50 and above. Furthermore, there was a statistically significant difference in the incidence of MB2 in maxillary second molars between patients aged below 40 and those aged 50 and above. The samples were then divided into two age groups, <50 and ≥50, and analyzed using a χ^2^ test. The test identified a statistically significant difference between the two age groups in the incidence of MB2 in the maxillary first and second molars (see Table 3).

**TABLE 3:** Comparison of the incidence of MB2 of maxillary molars between two groups divided by age.

Age	Tooth position	
	Maxillary first molar	Maxillary second molar
<50 years (852 teeth)	462 (54.2%)	303 (33.1%)
≥50 years (148 teeth)	49 (33.1%)	26 (17.6%)
Chi-square value	22.503	18.498
*p*	<0.001	<0.001

#### 3.2.4 MB root length

**FIGURE 2:**
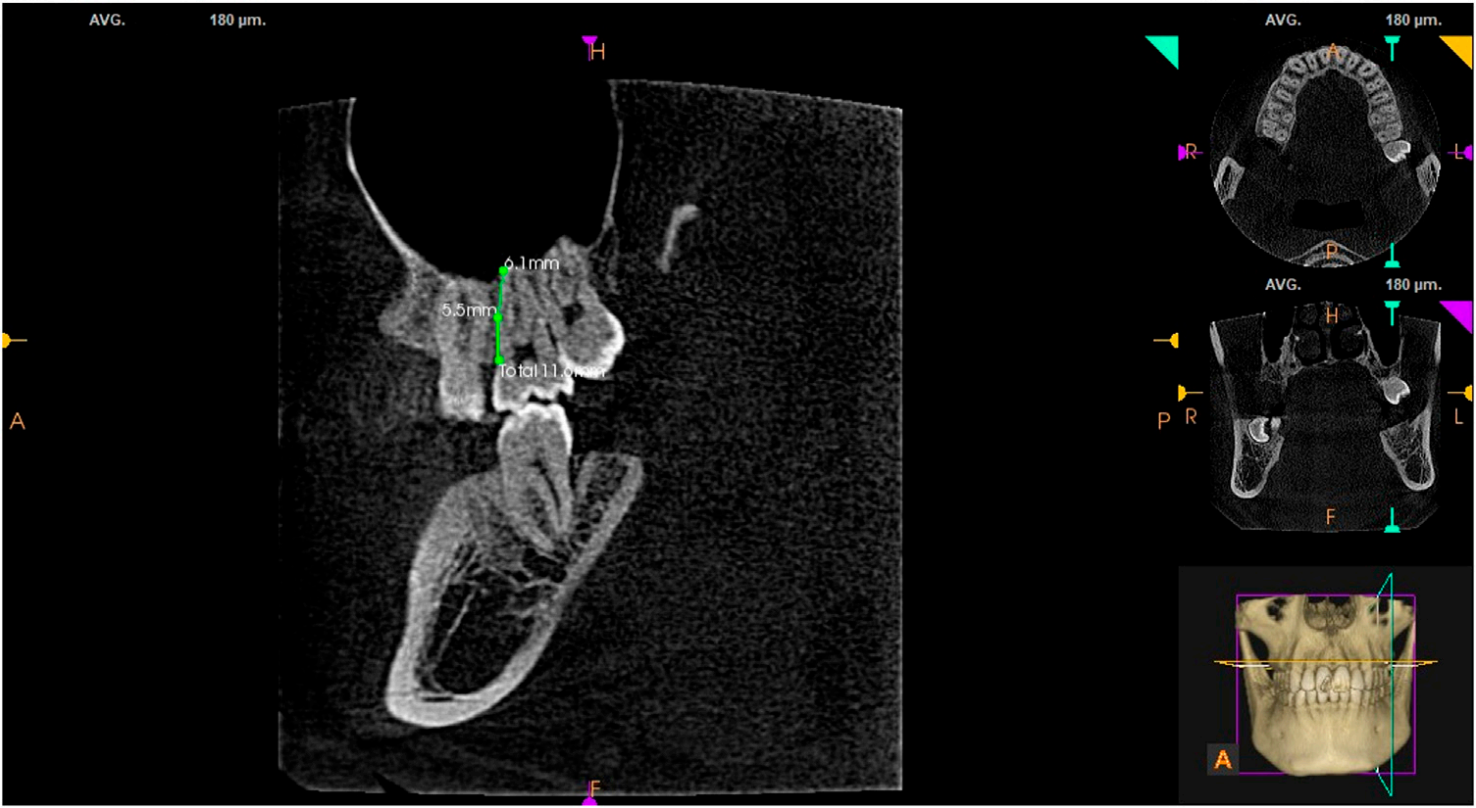
Length of MB root in maxillary second molars.

The MB root length was measured using the built-in tool of the CS 3D Imaging software by moving the roller continuously in the sagittal plane for observation, with reference to the distance from the cemento-enamel junction to the root apex (see Figure 2). It was found that the MB root length in the groups with and without MB2 in the right maxillary first molars was (12.05 ± 6.14) mm and (11.23 ± 1.22) mm, respectively, and the length in the groups with and without MB2 in the left maxillary first molars was (11.57 ± 1.38) mm and (11.08 ± 1.34) mm, respectively. The MB root length in the groups with MB2 in the right and left maxillary second molars was (12.46 ± 8.39) mm and (11.51 ± 1.32) mm, respectively, and the MB root length in the groups without MB2 in the right and left maxillary second molars was (11.44 ± 1.22) mm and (11.85 ± 4.38) mm, respectively. An independent sample rank-sum test carried out between the groups with and without MB2 indicated a significant statistical difference (*p* < 0.05; see Table 4).

**TABLE 4:** Comparison of the distance of MB root canal between groups with and without MB2.

		Tooth position			
		Tooth 16	Tooth 26	Tooth 17	Tooth 27
X ± S	With MB2	(12.05 ± 6.14)	(11.57 ± 1.38)	(12.46 ± 8.39)	(11.51 ± 1.32)
	Without MB2	(11.23 ± 1.22)	(11.08 ± 1.34)	(11.44 ± 1.22)	(11.85 ± 4.38)
Z		−3.593	−4.101	−2.907	−0.731
*p*		<0.001	<0.001	0.004	0.465

### 3.3 Partial anatomical features of the root canal system in maxillary first and second molars

#### 3.3.1 Classification of the MB root canal

Vertucci’s classification is the most widely applied and recognized method for classifying root canals (Vertucci, 1984; Tian et al., 2016). Statistical analysis found that the MB root in maxillary first and second molars was mostly a type I single root canal. It also found that MB2 was most commonly type IV, indicating that the main root canals of MB1 and MB2 are independent and separate single root canals extending from the pulp cavity floor to the root apex (2–2). Type II was the second most common type, indicating that the main root canals of MB1 and MB2 are independent root canals at the pulp cavity floor and become integrated as one root canal proximal to the root apex (2–1). Furthermore, the type of root canal in the left and right teeth of the same type was highly similar (see Table 5).

**TABLE 5:** The composition of MB in maxillary first and second molars in FuJian area.

Classification of root canal	16 (number of teeth/percentage)	Tooth 26	Tooth 17	Tooth 27
Type I	247 (49.4%)*	242 (48.4%)	337 (67.4%)^@^	331 (66.2%)^@^
Type II	51 (10.2%)	58 (11.6%)	48 (9.6%)	57 (11.4%)
Type III	6 (1.2%)	8 (1.6%)	22 (4.4%)	18 (3.6%)
Type IV	187 (37.4%)^#^	189 (37.8%)	84 (16.8)	86 (17.2%)
Type V	8 (1.6%)	3 (0.6%)	9 (1.8%)	8 (1.6%)
Type VI	1 (0.2%)			
Total (number of teeth)	500	500	500	500

*Mesiobuccal roots in maxillary first and second molars are typically type I.

#MB2 existed is typically type 2-2 with independent apical foramen.

@The type of MB root canal in left and right maxillary molars of the same name is highly symmetrical.

#### 3.3.2 Distribution characteristics of root canal orifices on the pulp chamber floor in maxillary first and second molars

**FIGURE 3:**
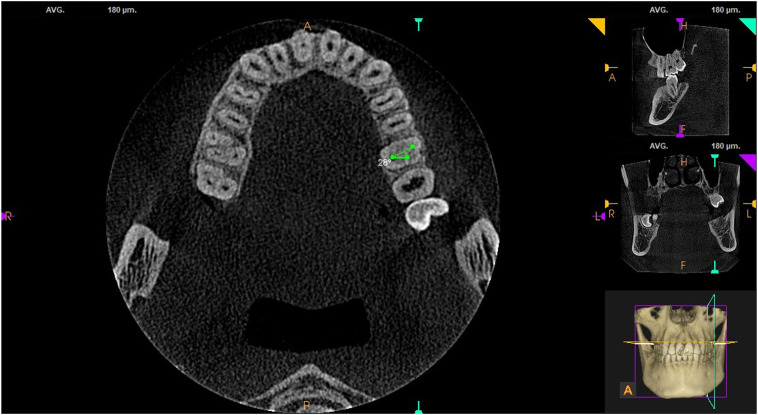
MB-P-DB on the pulp chamber floor in maxillary first molars.

Using the built-in measuring tool in the CS 3D Imaging software, connecting lines were drawn from fixed points at the center of the root canal orifices in the horizontal cross-section of the pulp chamber floor. The results showed that the angle formed by the MB-P-DB root canal orifices in maxillary first and second molars on the pulp chamber floor was (25.23 ± 5.20)° and (20.17 ± 10.88), respectively (see Figure 3).

## 4 Discussion

By summarizing and analyzing *in vivo* CBCT studies carried out in 21 regions, Martins (Martins et al., 2018) suggested that the global incidence of MB2 in maxillary first molars is 73.8%. However, it has been found that MB2 has a 72.4% missed detection rate, the highest among all types of root canal (Do Carmo et al., 2021). The incidence of MB2 may be affected by ethnicity, region, and study sample size, and it is closely related to the thickness of the CBCT scans used for observation. Bauman et al. identified that using scanning thicknesses of 0.4 mm and 0.125 mm resulted in an incidence of 60.1% and 93.3%, respectively (Bauman et al., 2011). The scanning thickness used in the present study was 0.18 mm, and the results identified the incidence of MB2 as 51.1% in maxillary first molars and 32.9% in maxillary second molars.

In a study of the factors influencing the incidence of MB2, Lin et al. found that maxillary molar MB2 mainly occurs in male patients (Tzeng et al., 2020). However, other researchers found no difference between genders in the incidence of MB2 (Reis et al., 2013), and the present study found that the incidence of MB2 in maxillary first and second molars was significantly higher in male patients than in female patients. Previous studies have shown that the calcification rate of the root canal in female patients is higher than that in male patients of the same age group (Turkal et al, 2013), which may explain why the incidence of MB2 in female patients in the present study was lower than that in male patients.

Regarding the effect of age on MB2, the present study found that there was a statistically significant difference in the incidence of MB2 between different age groups and that the incidence in patients below the age of 50 is significantly different from that in patients aged 50 and above. It is speculated that physiological changes associated with age, such as pulp cavity calcification and deposition of secondary dentin (Reis et al., 2013), may affect the detection of MB2. Whether the incidence is related to other factors, such as medication history, occlusal relationship, chewing habits, and the dietary preferences of patients in different age groups, is worthy of further in-depth study.

Whether there is a correlation between the incidence of MB2 in contralateral and ipsilateral maxillary molars is of great clinical significance. Tian (Tian et al., 2016) retrospectively analyzed CBCT images of 844 Chinese patients and obtained the following data: the probability of simultaneous presence of MB2 on both sides of the maxillary first molar was 79%, and the probability of the maxillary second molars as high as 82.3%, which was similar to the data obtained by Ratta (80.93% and 82.59%) (Ratanajirasut et al., 2018). Until now, there have been few studies on whether there is uniformity between the incidence of MB2 in maxillary first and second molars (Tzeng et al., 2020). The present study found a correlation between the incidence of MB2 in the left and right maxillary first and second molars in pairs, and the contralateral molars of the same type had a greater correlation than the ipsilateral adjacent molars. This finding can help clinicians become more vigilant and targeted in endodontic treatment.

Furthermore, the present study showed for the first time that MB root length in maxillary molars affects the incidence of MB2. For the four maxillary molars, according to the presence or absence of MB2, they are divided into two groups individually. The null hypothesis is that the MB root length distribution with and without the MB2 group is not statistically significant. Because the MB root length data does not follow the normal distribution, Mana-Whitney test was selected for statistical analysis of the data for each tooth position. The *p*-values corresponding to each tooth position were <0.001, <0.001, 0.004, 0.0465, the *p*-value for each group is less than 0.05, so the original hypothesis was overturned and it was considered that the MB root length distribution with and without MB2 group was statistically significant. Maxillary molars with MB2 have longer roots than those without MB2, and the intergroup difference is statistically significant. Navas reported in 2020 that the length of root passing through the buccal root of maxillary first molars is related to MB2 type, wherein short roots are typically type IV and long roots type II (Moidu et al, 2021). Whether these results are related to the activity of dentin deposition during root development requires further research at the genetic and clinical levels.

A previous CBCT study found that around a third of MB2 cases show an independent (2–2) apical foramen of type IV (Li, 2017), and the remaining MB2 and MB1 cases converge into the canal foramen of the same type (2–1). By classifying the MB root canal, the present study found that residents in Fujian, China, with MB2 mostly had an independent apical foramen, that is, their MB roots were mostly type IV (2–2), which is more likely than type II (2–1) to affect the success rate of root canal therapy in cases of missed diagnosis.

The regularity of root canal orifices on the pulp chamber floor has been used by many researchers to assist in the exploration and localization of MB2. Most researchers believe that the MB2 orifice is located mesial to the MB1-P line and on the palate side of MB1. According to Zhuk et al., the MB–MB2 distance is 2.06 ± 0.52 mm, and the distance between root canal orifices is typically greater in male patients than in female patients (Zhuk et al., 2020). This value is greater than that detected for maxillary molars in a Polish population, in which the MB–MB2 distance was 1 mm (Olczak and Pawlicka, 2017). Currently, the anatomical characteristics of the pulp chamber floor in maxillary molars are mostly limited to the distance and distribution direction of MB–MB2. In residents in Fujian, China, the angle formed by the MB-P-DB orifices on the pulp chamber floor in the maxillary first and second molars was (25.23 ± 5.20)° and (20.17 ± 10.88)°, respectively. In clinical practice, these data can be used to roughly locate the three main orifices in maxillary molars, thereby providing indirect assistance in the detection of MB2. There is no strong evidence to support its association with the incidence of MB2.

Although CBCT can assist in detecting root canals that are hard to find, this technique should be emphasized to apply reasonably and cautiously in ways that can maximize the interests of patients and minimize radiation exposure, as stipulated in the *Guidelines for radiographic examination in cariology and endodontics* (Society of Cariology and Endodontics and Chinese Stomatological Association, 2021).

## 5 Conclusion

This retrospective study found that the incidence of MB2 in maxillary molars was high in the Fujian province of China, especially in male patients below the age of 50. The length of the MB root was also found to influence the incidence of MB2. The anatomical morphology of root canals in the left and right maxillary molars is highly symmetrical. These finding help to provide imaging references for the clinical treatment of maxillary molar endodontic diseases in this region are of great clinical significance.

## Data Availability

The original contributions presented in the study are included in the article/Supplementary Material, further inquiries can be directed to the corresponding author.
